# Using histogram analysis of the intrinsic brain activity mapping to identify essential tremor

**DOI:** 10.3389/fneur.2023.1165603

**Published:** 2023-06-19

**Authors:** Pan Xiao, Li Tao, Xiaoyu Zhang, Qin Li, Honge Gui, Bintao Xu, Xueyan Zhang, Wanlin He, Huiyue Chen, Hansheng Wang, Fajin Lv, Tianyou Luo, Oumei Cheng, Jin Luo, Yun Man, Zheng Xiao, Weidong Fang

**Affiliations:** ^1^Department of Radiology, The First Affiliated Hospital of Chongqing Medical University, Chongqing, China; ^2^Department of Neurology, The First Affiliated Hospital of Chongqing Medical University, Chongqing, China

**Keywords:** essential tremor, machine learning, Radiomics, resting-state fMRI, amplitude of low-frequency fluctuation

## Abstract

**Background:**

Essential tremor (ET) is one of the most common movement disorders. Histogram analysis based on brain intrinsic activity imaging is a promising way to identify ET patients from healthy controls (HCs) and further explore the spontaneous brain activity change mechanisms and build the potential diagnostic biomarker in ET patients.

**Methods:**

The histogram features based on the Resting-state functional magnetic resonance imaging (Rs-fMRI) data were extracted from 133 ET patients and 135 well-matched HCs as the input features. Then, a two-sample t-test, the mutual information, and the least absolute shrinkage and selection operator methods were applied to reduce the feature dimensionality. Support vector machine (SVM), logistic regression (LR), random forest (RF), and k-nearest neighbor (KNN) were used to differentiate ET and HCs, and classification performance of the established models was evaluated by the mean area under the curve (AUC). Moreover, correlation analysis was carried out between the selected histogram features and clinical tremor characteristics.

**Results:**

Each classifier achieved a good classification performance in training and testing sets. The mean accuracy and area under the curve (AUC) of SVM, LR, RF, and KNN in the testing set were 92.62%, 0.948; 92.01%, 0.942; 93.88%, 0.941; and 92.27%, 0.939, respectively. The most power-discriminative features were mainly located in the cerebello-thalamo-motor and non-motor cortical pathways. Correlation analysis showed that there were two histogram features negatively and one positively correlated with tremor severity.

**Conclusion:**

Our findings demonstrated that the histogram analysis of the amplitude of low-frequency fluctuation (ALFF) images with multiple machine learning algorithms could identify ET patients from HCs and help to understand the spontaneous brain activity pathogenesis mechanisms in ET patients.

## Introduction

Essential tremor (ET) is one of the most common neurological disorders characterized by progressive tremor over time (1). The 2018 consensus statement of the Movement Disorder Society redefined ET as isolated bilateral upper limb action or postural tremor with a duration of at least 3 years (2). Meantime, ET accompanied by other neurological symptoms such as parkinsonism, ataxia, rest tremor, or non-motor symptoms has been relabeled as “ET plus” (2). This revised definition gave some advantages to including highly homogeneous ET patients for clinical trials. However, it was only based on clinical characteristics, and the etiology, pathology, biology, and pathogenesis of ET, especially the spontaneous brain activity changes, are still very unclear.

Resting-state functional magnetic resonance imaging (Rs-fMRI) is a frequently used non-invasive clinical imaging technique. The amplitude of low-frequency fluctuations (ALFF) as a reliable metric of Rs-fMRI that can detect the amplitude of spontaneously low-frequency oscillations in blood oxygen level-dependent (BOLD) signals is a promising way to explore spontaneous brain activity changes in many neuropsychiatric and neurological disorders, including ET (3–7). The results of recent studies that used ALFF analysis did generally vary, and most of these studies supported the view that ALFF changes in the cerebello-thalamo-cortical network (which is defined as the “classical tremor network”) were associated with ET patients (3, 8, 9). However, these studies were traditional mass univariate analyses, and they could not be used to predict ET patients at an individual level. Meanwhile, these ALFF analysis methods only used the average value of the ALFF image, and the ALFF image actually contained vast numbers of quantitative information, such as the histogram analysis features. Fortunately, these shortcomings can be remedied by radiomics analysis. This can abstract vast quantitative features including histogram information from ALFF images, and then these features are inputted to machine learning (ML) algorithms (10–13). ML builds the optimal models by learning and training from massive input data and then applies the model to new data to predict and analyze diseases based on a single-subject level (14–16). Moreover, ML-combined radiomics has shown to be a promising way to provide quantitative and objective supports for clinical diagnosis and prognosis and help to find a potential target for treatment, such as using ML algorithms based on diagnosis biomarkers from neuroimaging to identify ET from HCs to provide supporting evidence for clinically suspected ET diagnosis and guide the treatment of ET patients. Using ML algorithms combined with voxel-level local connectivity or frequency-dependent intrinsic brain activity analysis, our more recent studies revealed that these ML algorithms could achieve good classification performance to identify ET from healthy controls (HCs) (17–19). However, up to now, no studies have combined histogram analysis based on ALFF images of Rs-fMRI data with ML algorithms to identify ET patients.

Hence, the primary objective of the present study is to explore whether combined histogram analysis of ALFF images with multiple ML algorithms could be used to effectively distinguish ET patients and HCs. We also expected that our proposed method would not only reveal the intrinsic brain activity changes but also further act as the potential diagnosis biomarker in ET patients via the brain regions of the most power-discriminative features. Finally, we compared and contrasted the traditional univariate analysis with our proposed novel machine learning method to investigate whether the machine learning method was more sensitive than the univariate analysis and could overcome the intrinsic weaknesses of univariate analysis.

## Materials and methods

### Participants

This study was approved by the Ethics Committee of the First Affiliated Hospital of Chongqing Medical University in accordance with the Declaration of Helsinki. All participants were recruited from the First Affiliated Hospital of Chongqing Medical University and were required to sign the informed consent. The inclusion criteria of participants were the following: (1) All ET patients were diagnosed by two experienced neurologists (OM C, and Z X) according to the 2018 Movement Disorders Consensus Criteria; (2) the patients had an onset age between 18 to 55 years old, and patients with earlier or later onset were not included; (3) all participants were without thyroid disease, Parkinson’s disease, dystonia, psychogenic tremor, stroke, epilepsy, head injury, or any other neurological problems, and none of the HCs reported having relatives with ET; (4) all participants met the image quality and head motion control criteria (see Supplementary material). Finally, a total of 268 right-handed participants were recruited, including 133 ET patients and 135 age- and sex-matched HCs. In addition, demographic and clinical information was acquired before the completion of the MRI examination. The Fahn-Tolosa-Marin Tremor Rating Scale (TRS) and the Essential Tremor Rating Assessment Scale (TETRAS) were used to assess tremor severity and quality of life in ET patients. We also recorded the tremor frequency index from electromyography examination in ET patients. The Mini-Mental State Examination (MMSE), 17-item Hamilton Depression Rating Scale (HDRS-17), and Hamilton Anxiety Rating Scale (HARS-14) were used to briefly assess the cognitive function and mood status of all the participants, and we removed the patients with dementia (MMSE *<*24), depression (HDRS-17 *>* 7), and anxiety (HARS-14 *>* 7).

### MRI data acquisition

All MRI images were acquired using a GE Signa Hdxt 3.0-T MRI scanner (General Electric Medical Systems, Waukesha, WI) equipped with a standard 8-channel head coil. Rs-fMRI data were acquired using an echo-planar imaging (EPI) sequence with the following scan parameters: repetition time (TR) = 2000 ms, echo time (TE) = 40 ms, flip angle (FA) = 90°, 33 axial slices, slice thickness/gap = 4.0/0 mm, field of view (FOV) = 240 × 240 mm, matrix = 64 × 64, and a total of 240 volumes were collected which lasted 8 min. High-resolution 3D T1-weighted images were acquired using the following parameters: TR =8.3 ms, TE =3.3 ms, FA = 15°, FOV = 240 × 240 mm, matrix = 256 × 192, and slice thickness/gap = 1.0/0 mm. T2-weighted FLAIR images (TR = 8,000 ms, TE = 126 ms, TI = 1,500 ms, slice thickness/gap = 5.0/1.5 mm, FOV = 240 × 240 mm, and matrix = 256 × 192) were also acquired. During the scanning process, participants were told to keep their eyes closed, relax without actively thinking and stay awake in particular. Earplugs and foam padding were used to reduce scanner noise and minimize head movement.

### Data preprocessing and ALFF calculation

Data preprocessing was conducted using the Data Processing and Analysis of Brain Imaging toolbox (DPABI) (20), and detailed data-preprocessing steps were as follows: (1) Removal of the first 10 time points. For scanner stabilization and the acclimation of subjects to the MR scanning environment, the first 10 volumes were discarded, and the remaining 230 time points were included in the subsequent data preprocessing. (2) Slice timing correction. This was used to correct for a different acquisition time across slices in a volume. (3) Realignment. This was used to realign the subsequent functional images to the first volume to correct for within-run head motions, resulting in Friston 24 head motion parameters. These parameters were employed to assess the head movement and ensure the quality of Rs-fMRI data. (4) T1 segmentation and spatial normalization. The T1 images were co-registered to the mean Rs-fMRI data for each subject. Specifically, 3D T1-weighted images were segmented into gray matter (GM), white matter (WM), and cerebrospinal fluid (CSF) probability maps using SPM DARTEL segmentation. All the GM, WM, and CSF images were resampled to isotropic 1.5-mm voxels, spatially normalized to the MNI space using both affine transformation and non-linear deformation, and later, resampled to isotropic 3-mm voxel resolution with Rs-fMRI, and the deformation field was applied to the Rs-fMRI data. (5) Regression out Friston’s 24 head motion parameters and the mean time series of global, WM, and CSF signals. (6) Spatial smoothing with a Gaussian kernel of 4 mm full width at half maximum. (7) Detrending and filtering. These steps removed the extremely low-frequency drift and the high-frequency physiological noises. For detrending, we used first-order polynomial functions, and for filtering, we adopted band-pass filtering (0.01 Hz ~ 0.08 Hz) to the time series for each voxel. ALFF analysis was based on the pre-processed images. DPABI was also used to compute the ALFF as Zang et al. (4) described. Briefly, the filtered time series of each voxel was transformed into the frequency domain by the Fast Fourier Transform. Then, the power spectrums of the signal between 0.01 ~ 0.08 HZ were calculated, and the square roots of the power spectrums were ALFF values. Finally, for reducing the influence of individual ALFF variation and standardization purposes, the ALFF of each voxel was further divided by the global mean of ALFF values for each participant, and then individual smALFF maps were created of each subject for further analysis in our study.

### Abstraction of the histogram features

In this study, due to the key role of the cerebellum in the essential tremor, a structural-based atlas (an automated anatomical labeling atlas 3 (AAL3) with detailed subdivisions of the cerebellum) was applied for the Rs-fMRI data rather than a more adaptable functional-based atlas (Brainnetome atlas without the region of the cerebellum). Additionally, previous studies have demonstrated that some nuclei, such as the ventral posterior lateral nucleus of the thalamus and cerebellar dentate nucleus, were associated with tremor in ET patients (21–23). For the above-mentioned reasons, the AAL3 atlas with thalamus parcellation and combined bilateral cerebellar dentate nucleus was used to define the regions of interest (ROIs) in our study. However, some structures in the AAL3 altas (resolution: 1 × 1 × 1 mm) are so small (such as the nucleus reuniens of the thalamus and the right ventral tegmental area etc.) that they could not be identified in the Rs-fMRI images (resolution: 3 × 3 × 3 mm), and finally, only 159 ROIs were defined for extracting 15 intensity-based histogram features, including the mean, median, maximum, range, variance, skewness, kurtosis, 10th percentile, 90th percentile, inter-quartile range, mean absolute deviation, robust mean absolute deviation, root mean squared, energy, and total energy. We extracted a total of 2,385 histogram features from each participant’s mALFF images of Rs-fMRI data. The feature extraction procedure was performed using the open-source python package pyradiomics, and the description and formula of each feature can be found on their website.^1^

### Feature selection

Due to the curse-of-dimensionality or small-n-large-p problem (24), a total of 2,385 features greatly exceeded the sample size while most features were redundant and irrelevant. Therefore, feature selection is a necessary step to obtain the most important features and improve the accuracy of the model. Before the feature selection, the dataset was partitioned into training and testing sets in the ratio of 7:3, and a Z-score standardization was performed, respectively, to keep the data in sets mutually independent. Then, feature selection was conducted in the training set in three steps. Firstly, we performed a two-sample t-test on the 2,385 histogram features, and features with *p* < 0.01 were selected for the subsequent analysis. Then, the mutual information method (threshold = 0.1) was applied to further reduce dimensionality. Finally, we further applied a least absolute shrinkage and selection operator (LASSO) regression model to choose the most important features for classification (25). The LASSO performed both regularization and variable selection that compresses high-dimensional data by shrinking coefficients for weaker predictors toward zero and dropping variables from the model when their coefficients reach zero. A penalty term (|βi|) is added to the linear regression model in LASSO, which can shrink coefficients towards zero (L1 regularization). As the penalty term increases, the Lasso sets more coefficients to zero. The loss function of LASSO is as follows:

$$L=\overset{n}{\underset{i=1}{\sum }}{\left(y_{i}-{\hat{y}}_{i}\right)}^{2}+\lambda \overset{p}{\underset{j=1}{\sum }}\left|\beta_{j}\right|$$

The penalization parameter λ was tuned under the criteria of minimal mean squared error (MSE) to construct the optimal subset of features via a 10-fold cross-validated grid-search approach. Features with non-zero coefficients in the LASSO regression model were selected to train the classification model.

### Model construction and model evaluation

In our study, we constructed a nested loop to build the model; the outer loop was applied to evaluate the model performance by splitting the training and testing sets 100 rounds with a 7:3 ratio randomly, while the inner loop was used to determine the best parameters of classifiers via a grid search method with 10-fold cross-validation. The whole procedure of the nested loop is illustrated in Supplementary Figure S1. We used some common classifiers, the support vector machine with radial basis function kernel (RBF-SVM), logistic regression with the linear kernel (linear-LR, penalty = L2), random forest (RF), and k-nearest neighbor (KNN) to build models to differentiate ET and HCs based on the selected features. In the training set, a grid search method with 10-fold cross-validation was used to obtain the optimal parameters of each classifier (inner loop). After finding each optimal model, it was fitted to the entire training set and evaluated in the testing set, and this process was repeated 100 times (outer loop).

We took the mean value of accuracy (mACC), balance accuracy (mBACC), sensitivity (mSN), and specificity (mSP) into account to assess the classification results, while the mean receiver operating characteristic (mROC) curve was plotted to determine the performance of the classifiers, and the mean area under the curve (mAUC) was applied to evaluate the classification performance and validity of the diagnosis of these models. The model with the highest mAUC in the testing set will be considered the best model because the mAUC in the blind testing set will further reflect the ability of the classifier to generalize new and unseen data from independently recruited participants. In addition, permutation tests (1,000 times) were performed to test the significance of model performances by evaluating whether the accuracy and AUC were significantly higher than values by chance (14, 26).

### Identification of discriminative features

Due to the training set being slightly different in each outer iteration, the selected features were different, too. Therefore, we considered the features which were selected greater than or equal to 80 times in all 100 iterations as the most power-discriminative features and calculated the mean weights according to all outeriterations.

### Statistical analysis

We used SPSS to statistical process and analyze the demographic and clinical characteristics, the Kolmogorov–Smirnov test was used to assess the normality of continuous variables, the two-sample *t*-test was performed to analyze the normally distributed variables, the Mann–Whitney *U* test was used to analyze the non-normally distributed variables, and the chi-square test was applied to test the sex distribution. A partial Pearson’s correlation analysis was conducted to assess the relationships between the most power-discriminative features in the machine learning method and clinical tremor characteristics (e.g., tremor of onset, tremor of duration, tremor frequency, TRS-parts A&B, and TRS part C) in ET patients with the Bonferroni multiple corrections. A *p*-value <0.05 was considered statistically significant. Moreover, we performed classical univariate analysis for the mean ALFF values with the two-sample t-test, and the threshold was set at *p* < 0.01 with the Bonferroni multiple corrections.

## Results

### Demographic and clinical characteristics

In this study, 133 ET patients and 135 HCs were included. The demographic and clinical characteristics of all participants are summarized in Table 1. The demographic and clinical characteristics between the ET and HCs did not differ, except for the HARS-14 and MMSE scores, which were significantly different between the two groups (*p* = 0.0005 and *p* = 0.0034, respectively).

**Table 1:** Demographic and clinical features of ET and HCs.

Measure	ET	HCs	Statistics	*p* - value
Demographic				
Sample size	133	135	NA	NA
Age (years)	46.43 ± 14.14	44.58 ± 12.86	T = 1.12	0.2633
Gender (M:F)	67:66	78:57	*Z* = −1.21	0.2249
Education (years)	12.94 ± 4.47	12.25 ± 4.76	T = 1.22	0.2235
Handedness (R/L)	133:0	135:0	*Z* = 0.00	1.0000
Cigarette smoker	35/133	33/135	*Z* = −0.35	0.7253
Clinical of tremor				
Tremor of onset (years)	33.80 ± 10.60	NA	NA	NA
Tremor of duration (years)	12.62 ± 9.28	NA	NA	NA
Positive family history		NA	NA	NA
Positive	39	NA	NA	NA
Negative	94	NA	NA	NA
Alcohol sensitivity		NA	NA	NA
Positive	57	NA	NA	NA
Negative	41	NA	NA	NA
NA	35	NA	NA	NA
Tremor medication		NA	NA	NA
Propranolol	31(38.71 ± 18.48 mg)	NA	NA	NA
Tremor symmetry		NA	NA	NA
R = L	96	NA	NA	NA
R < L	11	NA	NA	NA
R > L	26	NA	NA	NA
Tremor frequency	6.96 ± 2.30	NA	NA	NA
TRS-parts A&B	23.31 ± 7.80	NA	NA	NA
TRS-part C	12.85 ± 6.83	NA	NA	NA
TETRAS	21.44 ± 7.14	NA	NA	NA
TET-ADSL	13.52 ± 7.19	NA	NA	NA
Clinical of psychology and cognitive				
HDRS-17	2.13 ± 1.17	2.20 ± 1.25	T = −0.44	0.6629
HARS-14	2.95 ± 1.19	2.28 ± 1.81	T = 3.51	0.0005
MMSE	28.66 ± 1.28	29.12 ± 1.25	T = −2.96	0.0034
Head movement				
FD_power	0.01 ± 0.06	0.01 ± 0.06	T = −0.09	0.9323
Scrubbing volumes	15.27 ± 7.95	15.77 ± 9.31	T = −0.47	0.6379

ET, essential tremor; HCs, healthy controls; HDRS-17, 17-item Hamilton Depression Rating Scale; MMSE, Mini-Mental State Examination; HARS-14, 14-item Hamilton Anxiety Rating Scale; TRS, Fahn-Tolosa-Marin Tremor Rating Scale.

### Classification performance

**Figure 1:**
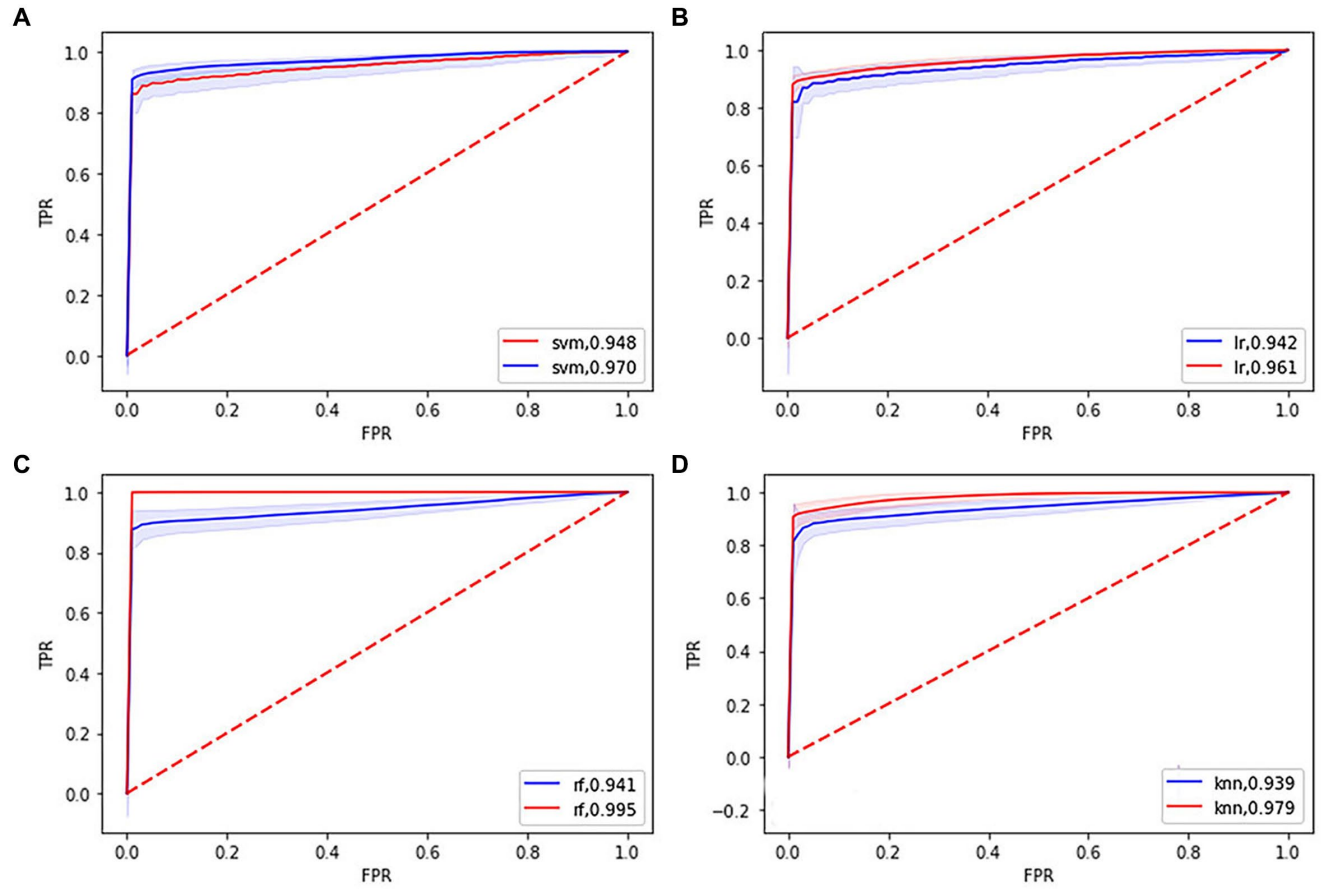
Receiver operating characteristic (ROC) curves and area under the curve (AUC) of four machine learning models. **(A)**, **(B)**, **(C)**, and **(D)** showing the ROC curves and AUC of the support vector machine, logistic regression, random forest, k-nearest neighbor model in the training (blue line), and testing set (red line), respectively.

Table 2 and Figure 1 show that the four classifiers including RBF-SVM, linear-LR, RF, and KNN achieved good classification performance in both the training and testing sets, implying the histogram radiomics analysis can effectively distinguish ET patients and HCs. In the training set, the mean accuracy and mAUC of the RBF-SVM, linear-LR, RF, and KNN are 94.87%, 0.970; 93.91%, 0.961; 99.49%, 0.995; and 95.16% 0.979, respectively. Despite the superior performance of all methods that we used in the training set, what really matters is the predictive results in the testing set. In the testing set, the mean accuracy and mAUC of the RBF-SVM, linear-LR, RF, and KNN are 92.62%, 0.948; 92.01%, 0.942; 93.88%, 0.941; and 92.27%, 0.939, respectively. The RBF-SVM classifier with the highest mAUC of 0.948 in the testing set was considered the best classifier, with mean accuracy and balance accuracy of 92.62 and 92.56%, respectively, mean sensitivity of 87.95%, and mean specificity of 97.17%. Permutation tests were performed in each testing set, and the results indicated the reliability of the observed accuracy and AUC, with all *p*-values <0.001 in iteration.

**Table 2:** The classification performance in the training set and testing set.

Method			Training set				Testing set			
	mAUC	mSN (%)	mSP (%)	mACC (%)	mbACC (%)	mAUC	mSN (%)	mSP (%)	mACC (%)	mbACC (%)
**SVM**	**0.970**	**89.91 ± 2.57**	**99.78 ± 4.68**	**94.87 ± 1.33**	**94.85 ± 1.34**	**0.948**	**87.95 ± 4.39**	**97.17 ± 3.71**	**92.62 ± 2.74**	**92.56 ± 2.74**
LR	0.961	88.47 ± 2.07	99.30 ± 0.80	93.91 ± 1.22	93.89 ± 1.22	0.942	87.17 ± 4.50	96.73 ± 3.44	92.01 ± 2.54	91.95 ± 2.55
RF	0.995	98.99 ± 1.31	99.99 ± 1.06	99.49 ± 0.66	99.49 ± 0.66	0.941	89.25 ± 4.35	98.39 ± 1.98	93.88 ± 2.44	93.82 ± 2.46
KNN	0.979	90.59 ± 4.66	99.68 ± 0.57	95.16 ± 2.41	95.14 ± 2.42	0.939	87.18 ± 4.48	97.24 ± 2.73	92.27 ± 2.39	92.21 ± 2.40

The values are expressed as mean ± standard deviation (`x ± s); AUC, area under the curve; SN, sensitivity; SP, specificity; ACC, accuracy; bACC, balance accuracy; SVM, support vector machine; LR, logistic regression; RF, random forest; KNN, k-nearest neighbor. The bold values means the best mAUC in the testing set in four classifiers.

### Discriminative features

**Figure 2:**
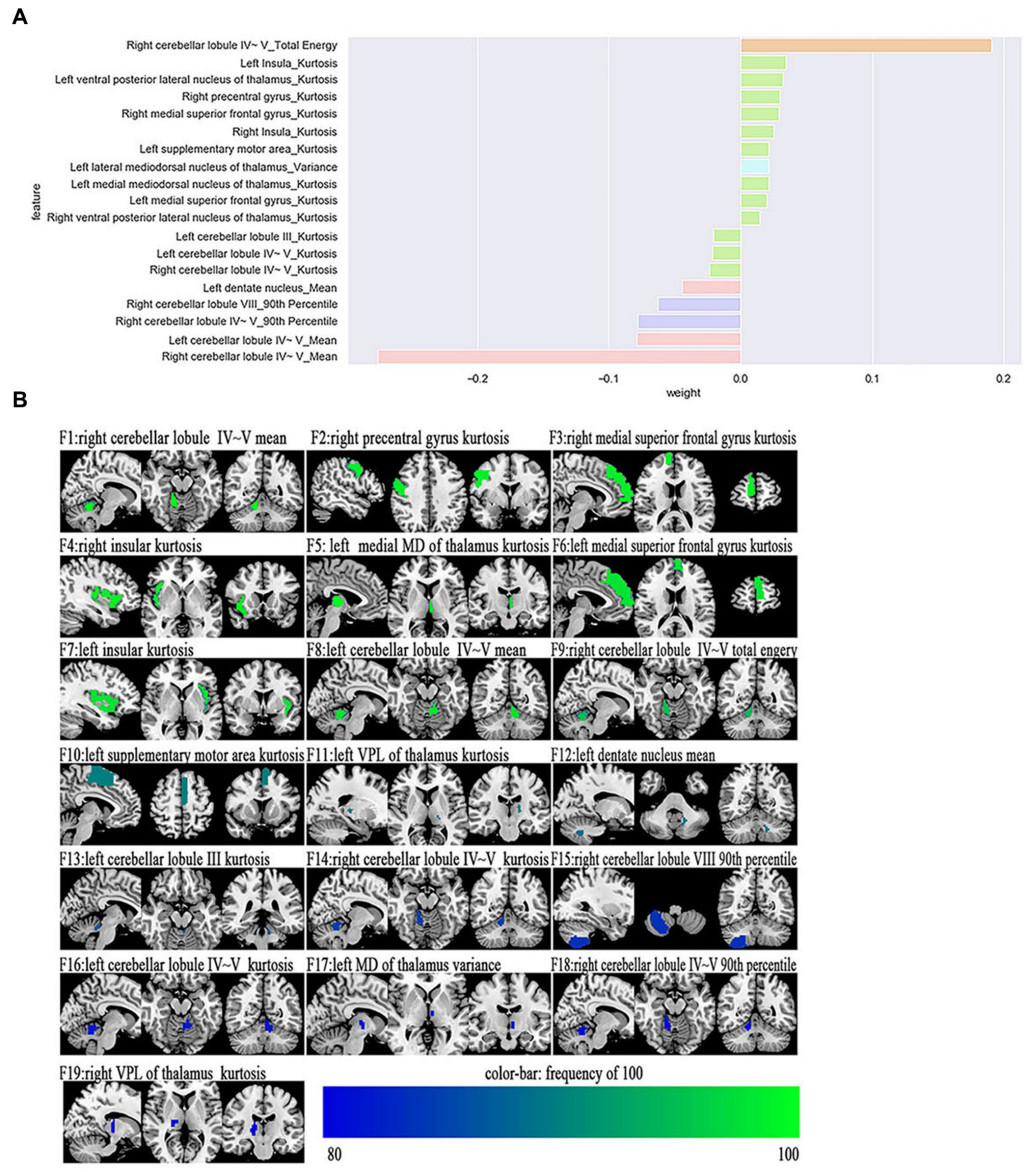
The selected most power-discriminative features. **(A)** shows the mean weight of the selected most power-discriminative features, and y-axis represents the most power-discriminative features, with their mean coefficients in the LASSO analysis plotted on the x-axis, of the same color mean the same type features. **(B)** shows the most power-discriminative features between ET and HCs groups, and the color bar value represents the frequency of the features.

The most power-discriminative features were identified as features that were repeatedly selected ≥80% in the outer iteration, and these features were considered to be helpful for ET classification. A total of 19 features are reported (Table 3; Figure 2), including the left cerebellar lobule III (Cerebellum_3_L)_kurtosis_, left cerebellar lobule IV ~ V with two features, (Cerebellum_4_5_L)_mean_, (Cerebellum_4_5_L)_kurtosis_, respectively; right cerebellar lobule IV ~ V with four features, (Cerebellum_4_5_R)_mean_, (Cerebellum_4_5_R)_total energy_, (Cerebellum_ 4_5_R)_kurtosis_, (Cerebellum_4_5_R)_90th percentile_, respectively; right cerebellar lobule VIII (Cerebellum_8_R)_90 Percentile_; left medial mediodorsal nucleus of the thalamus (Thal_MDm_L)_kurtosis_; left lateral mediodorsal nucleus of the thalamus (Thal_MDl_L)_variance_; left ventral posterior lateral nucleus of the thalamus (Thal_VPL_L)_kurtosis_; right ventral posterior lateral nucleus of the thalamus (Thal_VPL_R)_kurtosis_; right precentral gyrus (Precentral_R)_kurtosis_; left medial superior frontal gyrus (Frontal_Sup_Med_L)_kurtosis_; right medial superior frontal gyrus (Frontal_Sup_Med_R)_kurtosis_; left insula (Insula_L)_kurtosis_; right insula (Insula_R)_kurtosis_; left supplementary motor area (Supp_Motor_Area_L)_kurtosis_; and left dentate nucleus (dentate_L)_mean_.

**Table 3:** The significant discriminative features between ET and HCs.

AAL3 number	Features	AAL3 brain areas	Coefficient	Frequency
102	Total energy	Right cerebellar lobule IV ~ V	0.190466842	95
33	Kurtosis	Left insula	0.034522173	98
129	Kurtosis	Left ventral posterior lateral nucleus of the thalamus	0.032302957	90
2	Kurtosis	Right precentral gyrus	0.030070535	99
20	Kurtosis	Right medial superior frontal gyrus	0.029236559	99
34	Kurtosis	Right insula	0.025480698	99
15	Kurtosis	Left supplementary motor area	0.021742021	90
137	Variance	Left lateral mediodorsal nucleus of the thalamus	0.021271266	82
135	Kurtosis	Left medial mediodorsal nucleus of the thalamus	0.021267412	99
19	Kurtosis	Left medial superior frontal gyrus	0.019697482	98
130	Kurtosis	Right ventral posterior lateral nucleus of the thalamus	0.014803541	80
99	Kurtosis	Left cerebellar lobule III	−0.020707327	87
101	Kurtosis	Left cerebellar lobule IV ~ V	−0.021590975	82
102	Kurtosis	Right cerebellar lobule IV ~ V	−0.023882283	85
	Mean	Left dentate nucleus	−0.044511876	89
108	90th percentile	Right cerebellar lobule VIII	−0.062860398	84
102	90th percentile	Right cerebellar lobule IV ~ V	−0.078035109	81
101	Mean	Left cerebellar lobule IV ~ V	−0.078885995	98
102	Mean	Right cerebellar lobule IV ~ V	−0.275559008	100

AAL3, Automated Anatomical Labeling 3.

Additionally, relative to HCs, the ET group showed decreased ALFF values in the (Cerebellum_3_L)_kurtosis_, (Cerebellum_4_5_L)_kurtosis_, (Cerebellum_4_5_L)_mean_, (Cerebellum_4_5_R)_mean_, (Cerebellum_ 4_5_R)_total energy_, (Cerebellum_4_5_R)_kurtosis_, (Cerebellum_4_5_R)_90th percentile_, (Cerebellum_8_R)_90 Percentile_, and (dentate_L)_mean_, but increased ALFF values in the (Precentral_R)_kurtosis_, (Frontal_Sup_Med_L)_kurtosis_, (Frontal_Sup_Med_R)_kurtosis_, (Insula_L)_kurtosis_, (Insula_R)_kurtosis_, and (Supp_Motor_Area_L)_kurtosis_. Moreover, the thalamus of the ET group showed increased ALFF values in the (Thal_MDm_L)_kurtosis_, (Thal_MDl_L)_variance_, (Thal_VPL_L)_kurtosis_, and (Thal_VPL_R)_kurtosis_ compared to HCs (see Supplementary Table S1).

### Univariate analysis

The results of classical univariate analysis for the mean ALFF values with the two-sample t-test are reported in Supplementary Table S2. With a *p*-value <0.01, there were 24 features that showed significant group differences. However, only 11 features survived after the application of Bonferroni’s correction, including the bilateral cerebellar lobule III, cerebellar lobule IV ~ V, cerebellar lobule VI, cerebellar lobule VIII, left dentate nucleus, right ventral posterior lateral nucleus of the thalamus, and left lateral mediodorsal nucleus of the thalamus.

### Correlations between the discriminative features and clinical tremor symptoms

**Figure 3:**
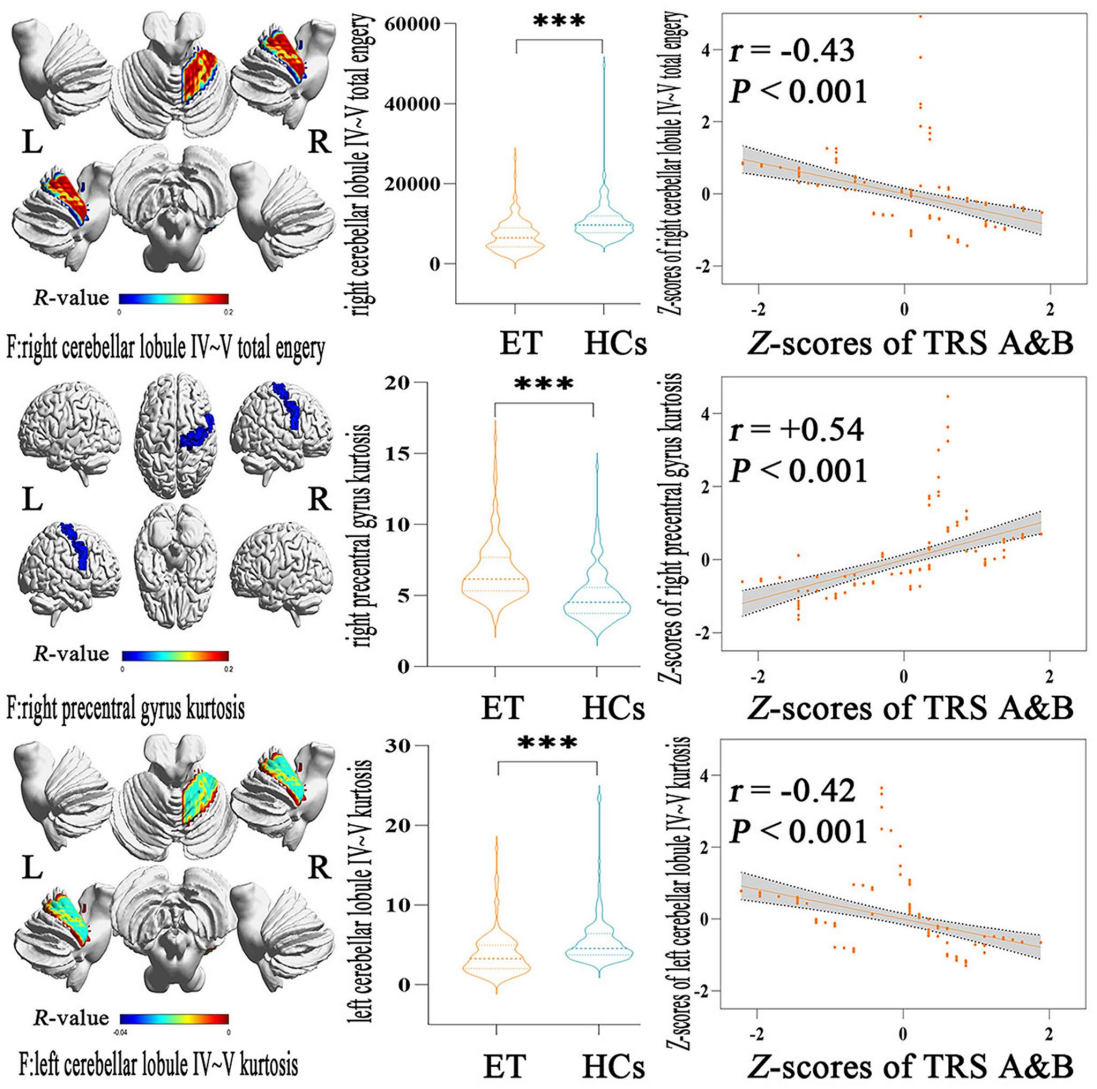
Partial Pearson Correlation analysis results between the selected histogram features and clinical tremor characteristics in ET patients. Bonferroni multiple comparison corrections, corrected *p* < 0.05/19^*^(19–1)/2. Violin plots displaying the mean and standard deviations of the selected histogram features in the ET and HCs group; scatter plots showing the correlation analysis in the ET group. ^***^p < 0.001. ET, essential tremor; HCs, healthy controls; zTRS A&B scores, z-transformed Fahn-Tolosa-Marin Tremor Rating Scale parts A and B scores.

Figure 3 showed the partial Pearson’s correlation analysis results, and 3 histogram features of ALFF parameters were significantly correlated with tremor severity in ET patients. The kurtosis of left cerebellar lobule IV ~ V and the total energy of right cerebellar lobule IV ~ V were negative correlation with TRS parts A&B (*p* < 0.001, *r* = −0.42 and − 0.43 respectively), and the kurtosis of right precentral gyrus was a positive correlation with TRS parts A&B (*p* < 0.001, *r* = 0.54).

## Discussion

In the present study, histogram features based on intrinsic brain activity mapping were combined with multiple machine learning algorithms to identify ET patients from HCs, and four main findings were reported. First, all of the four machine learning algorithms—RBF-SVM, linear-LR, RF, and KNN—achieved good classification performances; second, the most power-discriminative histogram features were mainly located in the cerebello-thalamo-motor and non-motor cortical pathways; third, some histogram features could be used to partially explain clinical tremor symptoms. Further, compared to univariate analysis, we found that machine learning with histogram features is more sensitive to intrinsic brain activity.

Histogram analysis as one of the most common radiomics methods is a newly emerging area in quantitative image analysis, and in this process, the medical images were divided into amounts of quantitative features. On account of this advantage, a few studies have achieved good classification performance for identifying Parkinson’s disease from HCs or Alzheimer’s Disease from HCs (12, 26–28). When combining clinical symptoms or brain gray matter volumes with machine learning algorithms, a few studies achieved good classification performance in classifying ET from HCs (29–32). Consistent with the above studies, our studies also achieved good classification performance. In fact, in the univariate analysis, we found that 11 mean ALFF features showed significant group differences with the application of strict Bonferroni’s correction, but only three discrimination features were mean ALFF values in the machine learning method, and the other 16 features were histogram analysis features including the total energy, kurtosis, variance, and 90th percentile. One reason is that the two-sample t-test was only the first step of feature selection in our machine learning method, and most features that remained in the univariate analysis did not survive in the LASSO regression model, and another reason is that the histogram analysis could give more quantitative information. Additionally, traditional univariate analysis is based on differences between the ET group and HCs group, and could not be used to predict ET patients at an individual level. These aspects further suggested that machine learning combined with histogram features allows the discovery of potential diagnosis biomarkers, predicts ET patients at an individual level, and may be more sensitive and accurate in revealing ALFF changes in ET patients, which overcomes the intrinsic weaknesses of univariate analysis.

Due to the benefits of predicting the individual subject and the multivariate nature of machine learning algorithms, radiomics analysis has been successfully used for neurological disease research and has provided quantitative and objective support for clinical diagnosis and disease prognosis (27, 33, 34). However, the clinical application of using machine learning algorithms to identify ET patients from HCs is limited. First, the generalizability should be further verified before the model is applied in clinical settings. Second, ET is a clinically evident condition that is easy to distinguish with HCs. Therefore, the expected purpose of this study is to reveal the intrinsic brain activity changes of ET and further act as the diagnosis biomarkers to identify ET from HCs. Third, misdiagnoses of ET are very common in clinical settings, such as being misdiagnosed as dystonic tremor, tremor-dominated PD, and even physiological tremors, etc. We hope to use the established diagnosis biomarkers of this study to differentiate ET with these disorders and reduce the misdiagnose rates in the future.

Using traditional ALFF analysis, very few studies revealed that ALFF changes in the classical tremor network were related to ET patients. However, these results were variable and even contradictory, and all of these studies did not reveal ALFF changes in the thalamus. Yin et al. (9) revealed that decreased ALFF in the cerebellum and increased ALFF in cerebral cortices were associated with ET patients. Li et al. (3) found a contradictory result, that increased ALFF in the cerebellum and decreased ALFF in cerebral cortices were involved in ET patients. However, our histogram analysis of ALFF values showed decreased ALFF values in the cerebellum and left dentate nucleus, with increased ALFF values in the cerebral cortices and the thalamus in the ET group. Our results seemed to be contradictory to Li et al. but in line with Yin et al. while also different in detail. We speculated that the following reasons may be reasonable explanations. First, ET is a kind of etiological, clinical, and pathological heterogeneity disease, and the heterogeneous properties may cause variable results from different researchers. Second, due to the small sample and absence of strict inclusion criteria, these results were more variable. Third, the ventral posterior lateral nucleus of the thalamus could not be identified in the common atlases, such as the anatomical automatic labeling atlas (AAL), Harvard Oxford atlas (HOA), and Brainnetome atlas (BNA), and these caused difficulty in directly revealing the ALFF changes in the classical tremor network. Finally, all the above studies also revealed ALFF changes in the cerebellar-cortical network. Therefore, our results were actually in line with the previous studies. Meantime, compared with the previous studies, a large sample size (133 ET patients and 135 HCs) and a strict inclusion (the 2018 Consensus Criteria of the Movement Disorder Society) were adopted in our studies. Meanwhile, the most power-discriminative features of histogram analysis changes were located in the classical tremor network, including the cerebellum, thalamus, and motor cortices. Growing evidence from histopathology, electrophysiological, neuroimaging, and neurobiology supported the view that the classical tremor network was associated with tremor in ET patients, and that especially the cerebellum played a vital role in tremor genesis. Very limited post-mortem studies showed that loss of Purkinje cells and changes in Purkinje cell morphology, including in the axonal and dendritic compartments, such as axonal swelling (torpedoes), thickened axonal profiles, increased recurrent axonal collaterals, axonal sprouting, and dendritic swelling, were the tissue-specific pathological characteristics of ET (35, 36). Evidence from electrophysiological assessments showed eyeblink conditioning was impaired in ET patients, an index of motor learning that relies on intact cerebellum (37). Moreover, using regional homogeneity, functional connectivity, or degree centrality analysis of Rs-fMRI (23), rhythmic finger tapping test fMRI (22), and positron emission tomography (PET) (38), studies also observed the central role of the cerebellum in the tremor genesis. Therefore, we suggest that the most power-discriminative features located in the classical tremor network further reinforced the classical tremor network pathogenesis theories, and decreased histogram analysis matrices in the cerebellum, such as those to assess total energy, kurtosis, mean and 90th percentile, which possibly reflect the primary pathological injury of the cerebellum in ET patients.

Moreover, most power-discriminative features were not only confined to the classical tremor network but also extended to the cerebello-non-motor cortical circuits, including the bilateral superior frontal gyrus and insula, and it seemed difficult for us to understand these aspects. First, growing evidence has indicated that ET is a syndrome caused by primary pathological damage in the cerebellum. The cerebellum has extensive connectivity with cerebral cortices including motor and non-motor cortices and regulates motor and non-motor functions. Second, strict inclusion criteria without gross cognitive impairment, depression, and anxiety were adopted to gain a highly homogeneous ET cohort in our study. The HARS-14 and MMSE scores were significantly different between the patients with ET and HCs in our study, and the differences between them are suggested to be based on confounding factors. However, all these ET patients could not meet the diagnosis of ET with anxiety and ET with total cognitive impairment according to the Diagnostic and Statistical Manual of Mental Disorders version four (DSM-IV) criteria and MMSE criteria, respectively. Additionally, we removed some ET patients with sub-threshold anxiety and total cognitive impairment (3 ET with HARS-14 scores in 6 and 4 ET with HARS-14 scores in 5, 2 ET with MMSE scores in 24 and 3 ET with MMSE scores in 25). We retained the HARS-14 and MMSE scores without significant difference between ET and HCs groups and repeated our study, and we gained similar results to before, with a good classification performance also showing the most discriminative features involved in cerebello-thalamo-motor and non-motor cortical pathways. Based on the above reasons, anxiety symptoms and cognitive status as confounding factors would not have changed the results in this study. Moreover, we acknowledge that the ET patients’ motor and non-motor symptoms were seen in the clinical setting, so our strict inclusion criteria could not remove the possibility of development of the above non-motor symptoms in the future or even remove a compensatory state to prevent the development of these non-motor symptoms.

## Limitations

There are several limitations to this study. First, although the relatively larger sample size and a good classification performance were achieved in our study, multi-center data would allow our results to be more generalization and stable in the future. Second, only histogram analysis of intrinsic brain activity mapping was selected as the input feature, and combined multimodal imaging data with clinical metrics would perhaps give more precise results in the future. Third, we reported the results using the structural-based AAL3 atlas rather than the functional-based Brainnetome atlas in the study based on Rs-fMRI data. A more adaptable functional-based atlas that contains the cerebrum and cerebellum could be used in the future to achieve further study aims. Finally, the diagnosis of ET relied only on clinical symptoms and neurological examinations. Due to the absence of diagnostic biomarkers and that misdiagnosis is common, we adopted strict enrollment criteria and annual follow-up to reduce misdiagnoses.

## Conclusion

In this study, combining histogram analysis of ALFF images with multiple machine learning algorithms achieved good classification performance for identifying ET patients from HCs. The most power-discriminatory features were not only confined to the typical tremor networks but also extended into non-motor networks, and these features can help to understand spontaneous brain activity pathogenesis mechanisms in ET patients.

## Data availability statement

The raw data supporting the conclusions of this article will be made available by the authors, without undue reservation.

## Ethics statement

The studies involving human participants were reviewed and approved by Ethics Committee of the First Affiliated Hospital of Chongqing Medical University. The patients/participants provided their written informed consent to participate in this study.

## Author contributions

PX and LT: conception, execution, design, execution, and writing of the first draft. XiZ, QL, HG, BX, XuZ, WH, HC, HW, FL, and TL: execution, review and critique. OC, JL, YM, and ZX: organization, review and critique. WF: conception, organization, design, execution, review and critique. All authors contributed to the article and approved the submitted version.

## Funding

This study was funded by the National Natural Science Foundation of China (NSFC: 81671663) and the Natural Science Foundation of Chongqing (NSFCQ: cstc2014jcyjA10047).

## Conflict of interest

The authors declare that the research was conducted in the absence of any commercial or financial relationships that could be construed as a potential conflict of interest.

## Publisher’s note

All claims expressed in this article are solely those of the authors and do not necessarily represent those of their affiliated organizations, or those of the publisher, the editors and the reviewers. Any product that may be evaluated in this article, or claim that may be made by its manufacturer, is not guaranteed or endorsed by the publisher.

## Footnotes

^1^ https://pyradiomics.readthedocs.io/en/latest/features.html
