# Supplementary material for: Using histogram analysis of the intrinsic brain activity mapping to identify essential tremor
Source: Front Neurol. 2023 Jun 19;14:1165603. doi: 10.3389/fneur.2023.1165603 (PMC10317178; doi:10.3389/fneur.2023.1165603)
Supplement: Supplementary file 1 [file Data_Sheet_1.DOCX]

**Using histogram analysis of the intrinsic brain activity mapping to identify essential tremor**

**Pan Xiao^†^, Li Tao^†^ , Weidong Fang^*^**

^†^These authors contributed equally to this work and share first authorship.

*******Corresponding author:**

Weidong Fang, Department of Radiology, The First Affiliated Hospital of Chongqing Medical University, No. 1 Youyi Road, Yuzhong District, Chongqing 400016, China. E-mail: [fwd9707@sina.com](mailto:fwd9707@sina.com)

Supplementary Material

1 Supplementary Material

1.1 image quality and head motion control criteria

All participants met the image quality and head motion control criteria. When we analyzed the RS-fMRI data, systematic tactics were carried out to deal with the major obstacle, non-neuronal physiological processes and head motion, of the intrinsic BOLD signal. First, the participants who had evidence of vascular or apparent structural brain defects on T2- or T1-weighted images, or whose T1-weighted images and the T2-weighted FLAIR images existed obvious gross brain structure or signals abnormalities were discarded by visual inspection, and no participants were removed. Second, the Friston 24 head motion parameters were regressed out, including six head-motion parameters (three translational and three rotational), six head-motion parameters one timepoint before, and the 12 corresponding squared items. Third, we regressed nuisance signals such as white matter (WM), cerebrospinal fluid (CSF), and also global signals. Fourth, we also deal with the volume-to-volume head motion, also called framewise displacement (FDs). Using mean FDpower > 0.2mm as a threshold (not FDJenk), the maximal scrubbings volumes were counted in our study, and two sample t-test was performed to explore whether these head parameters exist a significant difference between the two groups. The results showed that the maximal scrubbing volumes were 27 volumes (27/230 = 11.73%) in our study. The results showed that no significant difference in scrubbings volumes and the mean FDpower among the two groups was observed (scrubbing volumes:15.27 ± 7.95, 15.77 ± 9.31, *T* = -0.47, *P* = 0.6379; mean FDpower: 0.01 ± 0.06, 0.01 ± 0.06, *T* = -0.09, *P* = 0.9323).

2 Supplementary Figures

**Supplementary Figure 1: Schematic overview of the nested loop classification framework**
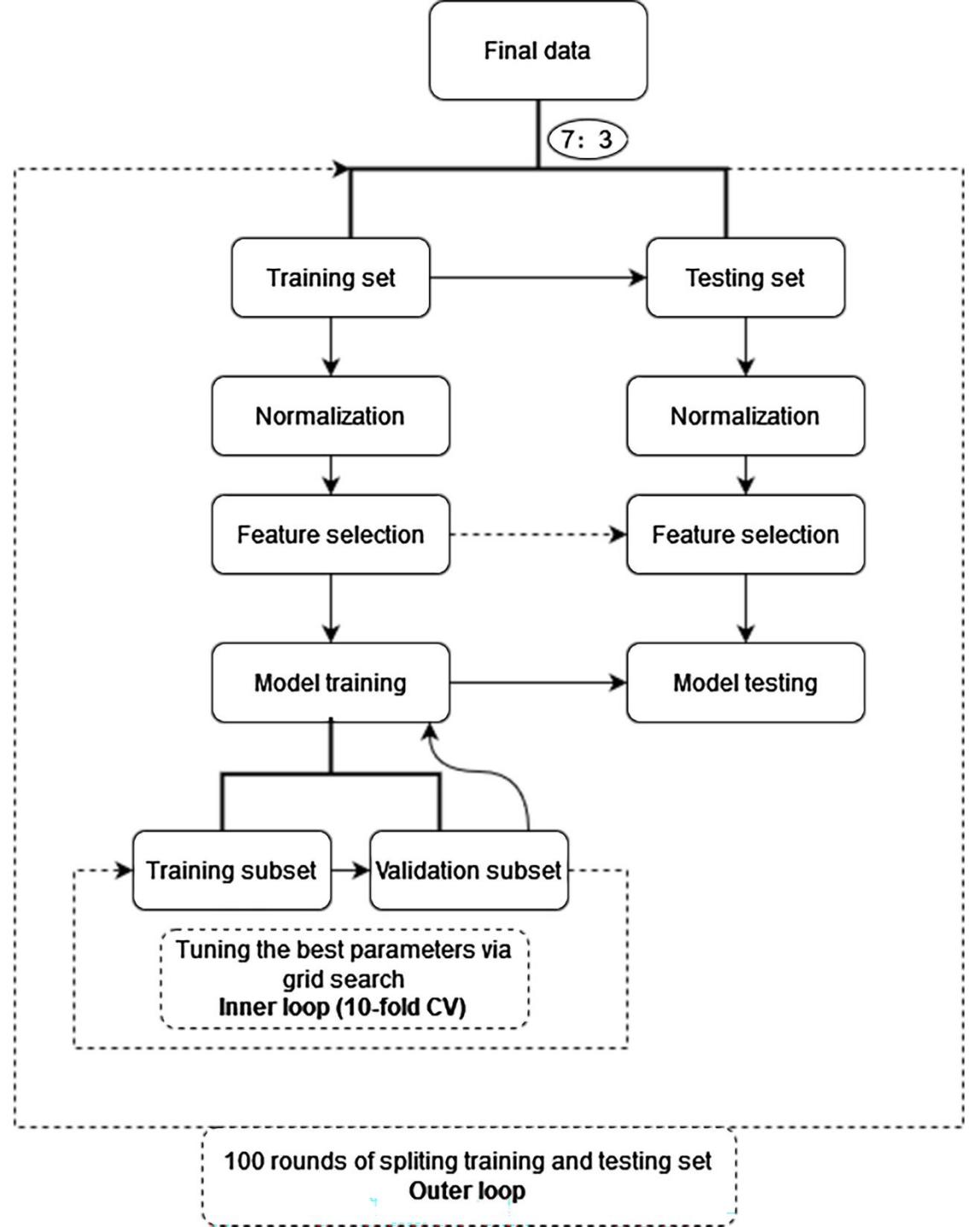


3 Supplementary table

**Supplementary table 1 Mean values and standard deviations of the most power discriminative features between ET and HCs in the machine learning method.**

|  | Features | AAL3 brain areas | HC | ET |
| --- | --- | --- | --- | --- |
| HC>ET | Kurtosis | Left cerebellar lobule IV~ V | 5.48±3.21 | 3.56±2.60 |
|  | Kurtosis | Right cerebellar lobule IV~ V | 5.42±2.53 | 3.62±2.78 |
|  | Kurtosis | Left cerebellar lobule III | 4.13±2.27 | 2.76±2.03 |
|  | 90th Percentile | Right cerebellar lobule IV~ V | 1.75±0.40 | 0.91±0.39 |
|  | 90th Percentile | Right cerebellar lobule VIII | 0.86±0.39 | 0.47±0.18 |
|  | Total Energy | Right cerebellar lobule IV~ V | 10445.21±4822.70 | 6562.99±3969.68 |
|  | Mean | Right cerebellar lobule IV~ V | 1.10±0.19 | 0.70±0.17 |
|  | Mean | Left cerebellar lobule IV~ V | 1.22±0.63 | 0.76±0.20 |
|  | Mean | Left dentate nucleus | 0.61±0.13 | 0.34±0.32 |
| ET>HC | Kurtosis | Left Insula | 6.03±4.96 | 11.10±5.91 |
|  | Kurtosis | Left ventral posterior lateral nucleus of the thalamus | 3.10±1.06 | 3.76±0.95 |
|  | Kurtosis | Right precentral gyrus | 4.95±1.80 | 6.87±2.04 |
|  | Kurtosis | Right medial superior frontal gyrus | 4.66±2.42 | 6.14±2.25 |
|  | Kurtosis | Right Insula | 5.44±3.43 | 8.62±4.21 |
|  | Kurtosis | Left supplementary motor area | 4.86±3.01 | 7.31±3.03 |
|  | Kurtosis | Left medial mediodorsal nucleus of the thalamus | 0.02±0.02 | 0.04±0.03 |
|  | Kurtosis | Left medial superior frontal gyrus | 4.109±1.61 | 5.88±2.59 |
|  | Kurtosis | Right ventral posterior lateral nucleus of the thalamus | 3.11±1.14 | 4.35±1.45 |
|  | Variance | Left lateral mediodorsal nucleus of the thalamus | 2.98±1.54 | 4.91±2.18 |
|  |  |  |  |  |

**Supplementary table 2 Mean values and standard deviations of the significant features between ET and HCs in the two-sample t-test. Statistical significance was set at p <0 .01 and Bonferroni correction for multiple comparisons was applied.**

|  | AAL  number | AAL3 brain areas | HC | ET | p |
| --- | --- | --- | --- | --- | --- |
| HC>ET | 99 | Left cerebellar lobule III | 1.24±0.35 | 0.79±0.36 | 0.000^*^ |
|  | 100 | Right cerebellar lobule III | 1.69±0.51 | 1.06±0.48 | 0.000^*^ |
|  | 101 | Left cerebellar lobule IV~ V | 1.22±0.23 | 0.76±0.20 | 0.000^*^ |
|  | 102 | Right cerebellar lobule IV~ V | 1.10±0.19 | 0.70±1.17 | 0.000^*^ |
|  | 103 | Left cerebellar lobule VI | 0.92±0.13 | 0.74±0.15 | 0.000^*^ |
|  | 104 | Right cerebellar lobule VI | 0.88±0.14 | 0.63±0.22 | 0.000^*^ |
|  | 107 | Left cerebellar lobule VIII | 0.64±0.19 | 0.39±0.13 | 0.000^*^ |
|  | 108 | Right cerebellar lobule VIII | 0.52±0.17 | 0.32±0.10 | 0.000^*^ |
|  | 145 | Left pulvinar medial of the thalamus | 0.79±0.15 | 0.75±0.13 | 0.009 |
|  |  | Left dentate nucleus | 0.61±0.13 | 0.34±0.32 | 0.000^*^ |
| ET>HC | 2 | Right precentral gyrus | 0.86±0.18 | 0.92±0.15 | 0.001 |
|  | 3 | Left dorsolatera of Superior frontal gyrus | 1.04±0.12 | 1.11±0.14 | 0.000 |
|  | 4 | Rightt dorsolatera of Superior frontal gyrus | 1.12±0.14 | 1.19±0.16 | 0.000 |
|  | 15 | Left supplementary motor area | 1.12±0.09 | 1.22±0.09 | 0.000 |
|  | 16 | Right supplementary motor area | 0.94±0.16 | 1.02±0.16 | 0.000 |
|  | 33 | Left Insula | 0.81±0.09 | 0.85±0.09 | 0.001 |
|  | 129 | Left ventral posterior lateral nucleus of the thalamus | 0.71±0.10 | 0.76±0.09 | 0.000 |
|  | 130 | Right ventral posterior lateral nucleus of the thalamus | 0.65±0.10 | 0.87±0.08 | 0.000^*^ |
|  | 135 | Left medial mediodorsal nucleus of the thalamus | 1.07±0.18 | 1.17±0.18 | 0.000 |
|  | 136 | Right medial mediodorsal nucleus of the thalamus | 1.01±0.17 | 1.10±0.15 | 0.000 |
|  | 137 | Left lateral mediodorsal nucleus of the thalamus | 0.94±0.18 | 1.04±1.07 | 0.000^*^ |
|  | 138 | Right lateral mediodorsal nucleus of the thalamus | 0.90±0.17 | 0.97±0.15 | 0.000 |
|  | 151 | Left subgenual of anterior cingulate cortex | 1.50±0.61 | 1.77±0.49 | 0.000 |
|  | 152 | Right subgenual of anterior cingulate cortex | 1.05±0.40 | 1.24±0.37 | 0.000 |
|  |  |  |  |  |  |

AAL3: Automated Anatomical Labeling 3

*: The feature survived after the application of Bonferroni’s correction
